# Improved growth of pea, lettuce, and radish plants using the slow release of hydrogen sulfide from GYY-4137

**DOI:** 10.1371/journal.pone.0208732

**Published:** 2018-12-17

**Authors:** Justin M. Carter, Eric M. Brown, James P. Grace, Aliasger K. Salem, Erin E. Irish, Ned B. Bowden

**Affiliations:** 1 Department of Chemistry, University of Iowa, Iowa City, Iowa, United States of America; 2 College of Pharmacy, University of Iowa, Iowa City, Iowa, United States of America; 3 Department of Biology, University of Iowa, Iowa City, Iowa, United States of America; University of Calgary, CANADA

## Abstract

Hydrogen sulfide (H_2_S) is a key gasotransmitter in agriculture and has been reported to increase the growth of plants in the first two weeks and to mitigate the effects of environmental stressors. GYY-4137 is widely used in these studies because it slowly releases H_2_S, but there is disagreement as to whether it requires enzymes to release H_2_S. In this article we describe the release of H_2_S in water without enzymes and that it releases H_2_S faster in organic solvents than in water or when mixed in topsoil. Furthermore, we describe the long-term effect of dosing pea, radish, and lettuce plants with GYY-4137 for up to six weeks. The effect of GYY-4137 on plant growth for six weeks was either positive or negative depending on the loading of GYY-4137 and how it was applied to plants. The addition of GYY-4137 to lettuce plants via potting mix resulted in reduced growth and death of the plants. In contrast, application of GYY-4137 to the leaves of lettuce plants increased the harvest weight of the leaves by up to 86%. Our results demonstrate that GYY-4137 can have a positive, important effect on the growth of plants but that this effect is dependent on several factors.

## Introduction

The worldwide population is expected to grow from its current level of 7.2B to 9.6B people by 2050.[1–3] To meet the needs of the world’s growing human population, it is projected that global food production must increase 70% by 2050.[4] Aside from sheer population numbers, there are additional factors that will increase market demand for food production. Trends indicate that as developing countries urbanize and their economies grow, their consumption of meat and dairy products will also increase. Because the conversion of feed to livestock is inefficient (for instance, each pound of hamburger requires 52 pounds of feed grain over a cow’s lifetime), [5] growth in this sector creates an increasing demand for cereal crops. Biofuel production may drive cereal demands even higher. Yet the amount of land dedicated to farming has remained at 1992 levels both in the United States and worldwide, which has led to a decrease in agricultural area per capita from 0.44 hectares per capita in 1960 to 0.17 hectares per capita in 2025.[6, 7] Current advances in technology cannot increase the yield per acre of crops enough to feed the growing population; new innovations are needed. Solutions must be environmentally safe, nonpolluting, and should help crops survive droughts and other environmental stressors that affect their growth.

One partial solution to this problem is the application of hydrogen sulfide (H_2_S) to increase the growth, survival, and yields of crops. H_2_S is a gasotransmitter that is synthesized enzymatically in plants and used as a signaling molecule. Research in the last dozen years has repeatedly demonstrated that therapeutic amounts of H_2_S have dramatic effects, including increased growth of roots, protection against heat stress and drought conditions, increased overall size and mass, alleviation from freezing stress on leaves, protection from high water salinity, and prolonged fruit shelf life.[8–13] The investigation of H_2_S in plants is a new field–much of the key work has been completed since 2007 –yet it has already been shown to have positive effects on corn, soybeans, wheat, sweet potatoes, cucumbers, strawberries, rice, spinach, tomatoes, broccoli, and kiwi.[14–39] In this publication we report how GYY-4137, which slowly releases H_2_S by hydrolysis, increased the growth of radish, peas, and lettuce plants. We report that the harvest yield of radishes doubled when milligram loadings of GYY-4137 were applied.

**Fig 1:**
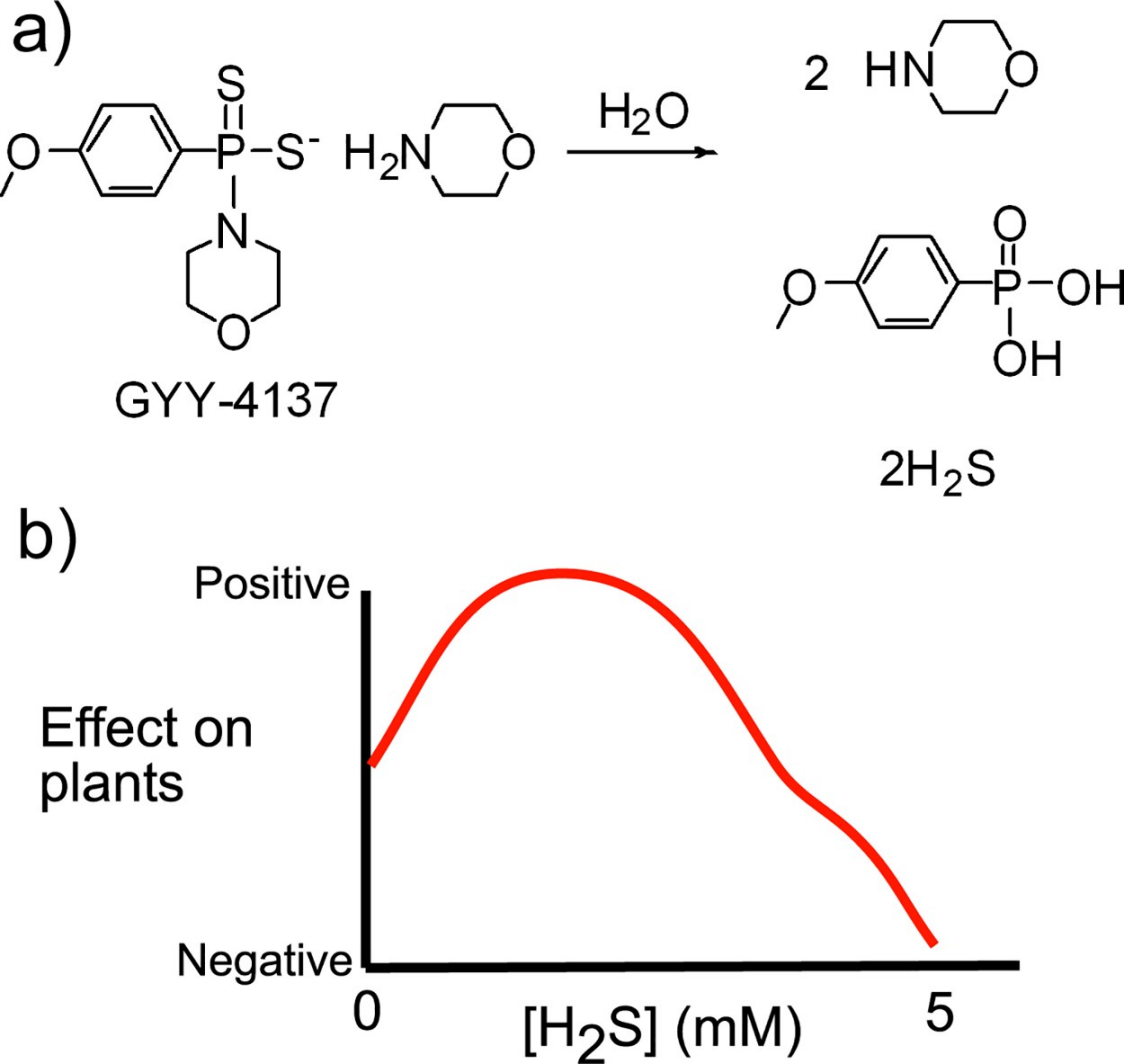
a) GYY-4137 hydrolyzes to release H_2_S and other chemicals. b) A schematic of the general effect that exogenous H_2_S has on plant growth is shown. The concentrations of aqueous H_2_S that have a negative effect on plants differs based on how often the plants are dosed and which effect is being investigate, 5 mM of aqueous H_2_S leads to negative effects on most plants.

Two of the challenges of working with H_2_S is that it is a low boiling point gas (boiling point = -60°C), and it is highly toxic. Exposure to levels of 2 ppm of H_2_S in the air can lead to negative health effects such as headaches or breathing problems for people who suffer from asthma, and exposure to 100 ppm is “immediately dangerous to life and health”.[40] Since H_2_S is challenging to handle, most scientists who study it add solid NaSH to water to yield an aqueous solution of H_2_S. These aqueous solutions can be used to deliver H_2_S, but they also lead to a rapid release of H_2_S into the atmosphere. Much prior work to investigate the effect of H_2_S in crops involved dosing plants or seeds daily with aqueous solutions of H_2_S at levels of 10 μM to several mM, which led to rapid evaporation of H_2_S and the potential release of >100 ppm of H_2_S into the atmosphere. The low boiling point of H_2_S and its high toxicity places obvious limitations on how aqueous H_2_S can be used to grow crops. To address this challenge, chemicals such as GYY-4137 that slowly release H_2_S were used to investigate the effect of exogenous H_2_S on plants (Fig 1).[41–45] The use of these chemicals minimizes the levels of H_2_S in the atmosphere above plants and can localize the release of H_2_S to specific parts of plants. Despite the widespread use of GYY-4137 in cell studies and agriculture, there remains some confusion about whether its release of H_2_S is driven primarily by hydrolysis in water or whether it is enzymatic.[46–48]

Prior work in the field of positive effects of H_2_S on plants involve studies of seedlings or how young plants survive environmental stressors. These studies typically reported a bell-shaped response curve such as that in Fig 1B. At high concentrations of H_2_S plants do not survive, but at intermediate loadings the effect of H_2_S was positive for numerous characteristics of plants.[8, 11, 14, 49–54] The concentration of H_2_S where the therapeutic effect was observed differed for each set of plants. Numerous studies reported that when seeds were grown hydroponically with H_2_S at concentrations of 10 μM to 1 mM, the rate of germination and growth of plants improved but higher concentrations of H_2_S caused the seeds to germinate slowly or not at all.[29, 34, 55–59] Other articles report that when plants were grown for a few days to a few weeks with exposures to different levels of H_2_S and then exposed to environmental stressors such as drought, flood, high arsenic levels in the water, or low Fe levels, those that were initially exposed to intermediate loadings of H_2_S had higher survival rates compared to plants not exposed to H_2_S.[34, 46, 50, 52, 54, 56, 59, 60]

What is mostly missing in this field are long term studies of the effect of H_2_S on the growth of plants and the effect of H_2_S on harvest yields. The only reports of exogenous H_2_S on harvest yields were studies in 1978 and 1979 where plants were grown in greenhouses for months with constant levels of 0 to 300 ppb of gaseous H_2_S.[61, 62] As a point of calibration, humans can smell H_2_S to concentrations as low as 500 ppb although a more common lower level for human smell is 1,000 ppb. At intermediate loadings of 30 to 100 ppb H_2_S, lettuce heads increased in weight by up to 47%, sugar beet roots increased in weight by up to 69%, and the weight of cotton flowers and bolls increased by up to 177%. Despite these initial promising results, recent work has focused on the effect of H_2_S on first couple weeks of growth of plants or their response to environment stressors.

In this article we report on the effect of H_2_S on the growth of pea and lettuce plants for 3 to 6 weeks, and we report on the growth and harvest yields of radishes. These plants were chosen as the edible portions are different: the leaves of lettuce, the fruits and seeds of peas, and the roots of radishes. Plants were grown with different levels of GYY-4137 administered in the soil (for all plants) or onto the leaves (lettuce) to investigate the effect of how different loadings of GYY-4137 and different locations of applications had on their growth. Furthermore, we report on the hydrolysis of GYY-4137 in water and organic solvents and how soil can affect its hydrolysis.

Little work has been reported on the effect of aqueous H_2_S or GYY-4137 on pea, radish, or lettuce plants. Previously mentioned articles in 1978 and 1979 used gaseous H_2_S and reported surprisingly large effects on the growth of lettuce plants, but this work was not further explored for over three decades. A paper in 2015 reported the effect of growing lettuce seeds for 5 days in water with different concentrations of H_2_S.[51] Seeds grown in 0.01 mM H_2_S had a 40% increase in elongation compared to control plants grown without H_2_S. This effect lessened at concentrations of 0.1 and 0.5 mM H_2_S, and plants grown in 1.0 mM H_2_S had over 50% reduction in elongation compared to the control plants. A paper in 2014 reported that low levels of aqueous H_2_S slowed senescence of harvested lettuce for 48 h.[12] Two papers in 2013 and a third in 2015 report on the effect of exogenous H_2_S on pea plants.[50, 58, 63] The growth of pea seeds using hydroponics with different levels of H_2_S in the water led to an increase in size of 68% for pea plants grown in 100 μM H_2_S for 5 days compared to control plants grown in the absence of H_2_S. Young pea plants exposed to H_2_S and either arsenate or flooding had higher survival rates compared to plants that were not exposed to H_2_S. Finally, the only reports of the effect of H_2_S on radish plants were published in the 1990s and report that all loadings of H_2_S had a negative effect on these plants.[64–66] None of the prior work on peas, lettuce, and radishes investigated the effect of aqueous H_2_S for longer than a week or any effect on yield.

## Experimental

### Material and methods

Plants were grown in a 1:1 mixture of #1 Professional Germination Mix and #4 General Purpose Growing Mix, Beautiful Land Products of Iowa LLC in West Branch, Iowa, prewet before loading pots to maximize consistency. Lawesson’s reagent, morpholine, bis(2-hydroxyethyl)amino-tris(hydroxymethyl)methane (BIS-TRIS), and all solvents used were obtained from Sigma Aldrich. GYY-4137 was synthesized from a published method.[67] Lettuce cv. Grand Rapids TBR variety purchased from Earl May. Pea cv. Green Arrow and radish cv. Cherry Belle radishes purchased from Eden Brothers. Seeds were sown in 4” square pots (538 cm^3^ potting mix) or 2.5” square pots (232 cm^3^ potting mix). Plants were grown on a rooftop with watering as needed.

### Administration of GYY-4137

GYY-4137 was administered to the seeds using four different methods. In method 1, enough potting mix to fill 12 pots was added to a larger bin and watered to give a consistent wetness. The GYY-4137 was added to the potting mix and blended thoroughly by hand until it was evenly distributed. Finally, 12 pots were filled with the potting mix and packed finger tight. In method 2 GYY-4137 was added to freshly planted seeds as a solution. In this method seeds were sown approximately 2 cm deep and covered with potting mix. Enough GYY-4137 for 12 plants was added to 60 mL of tap water and vigorously stirred. Five mL of this aqueous solution of GYY-4137 was pipetted into the soil directly above the seeds.

In methods 3 and 4 the plants were dosed with GYY-4137 at planting (day 0) and subsequently every 7 days. In method 3 the seeds were initially planted according to method 2, in which aqueous GYY-4137 was added to the potting mix above the seed immediately after planting. Every 7 days enough GYY-4137 for 12 plants was added to 60 mL of water and vigorously stirred. The aqueous GYY-4137 was added to the potting mix immediately adjacent to the leaves of the plant. In this method plants were dosed with the same amount of GYY-4137 every 7 days as they were dosed at day zero. For instance, if a plant was originally dosed with 10 mg of GYY-4137 at day zero, it was dosed with 10 mg of GYY-4137 every 7 days. In method 4, used for lettuce only, after planting according to method 2, leaves were treated weekly with an application of dosing of GYY-4137. Every 7 days enough GYY-4137 for 12 plants was added to 60 mL of tap water and vigorously stirred. Five mL of aqueous GYY-4137 was added to the shoot tip of each plant in the amount added to the soil above the seeds at day zero.

### Planting and watering of seeds

For each set of plants and each method of delivering GYY-4137, twelve seeds were planted at each loading of GYY-4137. For instance, radish plants were grown using methods 1 and 2. For each method radish seeds were exposed to eight different loadings of GYY-4137, and twelve radish seeds were planted at each loading for a total of 96 radish seeds sown for method 1 and 96 seeds for method 2.

Lettuce seeds were sown in 2.5” pots about 2 cm deep. The lettuce plants were grown in a greenhouse located at the University of Iowa Biology building. Lettuce seeds were sown on June 15, 2017 and harvested on July 6 for the 3-week harvest and July 27–28 for the 6-week harvest. Pea seeds were planted about 2 cm deep in 4” pots. The pea plants were grown outside from June 23, 2017 and harvested on July 17 for 3-week harvest. The 6-week harvest was done on August 3 and 4. Radish seeds were planted 2 cm deep in 2.5” pots. The radish plants were grown outside from June 30, 2017 to July 31, 2017. All the plants were watered once daily or as needed.

### Plant harvesting and quantification

Radishes were harvested at 4.5 weeks. After cutting off the shoot and brushing off the potting mix, roots were weighed on a laboratory balance. Pea plants were harvested at both 3 and 6 weeks by cutting the plant at the root/shoot boundary and removing the leaves of the plant. The height of the shoot was measured from the root/shoot boundary to the base of the uppermost expanding leaf. The shoot (stem plus detached leaves) was then weighed on a laboratory balance. Lettuce plants were harvested at 6 weeks by cutting the stem at the root/shoot boundary. Soil was gently brushed from the plants, and the total shoot weight was obtained by weighing the leaves and the stem. The leaf weight was determined by cutting the leaves from the stem and weighing the leaves separately.

### Qualitative H_2_S detection with lead acetate strips

H_2_S release from aqueous solutions of GYY-4137 at 0.5 M was assayed using lead acetate strips that turn from white to brown and then black when exposed to H_2_S. GYY-4137 was added to BIS-TRIS buffered to pH 7 to ensure a constant pH. In each of the experiments either 0.6 mL (subscript 1) or 1.2 mL (subscript 2) of buffered GYY-4137 was used. Aqueous GYY-4137 was added to a 20 mL scintillation vial and a separate, smaller vial which contained a lead acetate strip was placed into the 20 mL vial. The lead strip did not come in contact with the aqueous GYY-4137 but H_2_S released by GYY-4137 partitioned into the atmosphere and contacted the strip. The 20 mL vial was capped tightly and further sealed with parafilm. The vials were kept at room temperature for 10 days.

In samples labelled A_1_ and A_2_ the lead strips were exposed to aqueous GYY-4137. In samples labelled B_1_ and B_2_ the GYY-4137 was replaced with NaSH (also at 0.5 M) as a positive control for H_2_S release. In samples labelled C_1_ and C_2_ potting mix used here was added to the 20 mL vial with aqueous GYY-4137. Samples labelled D_1_ and D_2_ were identical to C_1_ and C_2_ except that NaSH was used in place of GYY-4137. For samples labelled E_1_ and E_2_ locally collected topsoil was added to the aqueous solution of GYY-4137. Samples labelled F_1_ and F_2_ were identical to E_1_ and E_2_ except that NaSH was used rather than GYY-4137.

### ^31^P NMR spectroscopy investigation of hydrolysis of GYY-4137

The hydrolysis of GYY-4137 was monitored in 90% H_2_O/D_2_O at room temperature by ^31^P NMR spectroscopy. Two studies were performed at 0.12 M and 0.50 M GYY-4137 to investigate the rate of hydrolysis above and below the solubility limit of GYY-4137 in water (0.13 M). GYY-4137 was dissolved in 1.5 mL of 90% H_2_O/D_2_O buffered with BIS-TRIS (1 M) at pH 7. The ^31^P NMR spectra were collected at day 0 and at day 35. The NMR spectra were obtained using a Bruker Avance-300 at 300-MHz, Bruker DRX-400 at 400-MHz, and Bruker DPX-500 at 500-MHz. The hydrolysis of GYY-4137 was also measured in DMSO-d_6_ at a concentration of 0.13 M. The ^31^P NMR spectra were collected at day 0 and periodically after that time.

### H_2_S detection with H_2_S electrode

H_2_S detection with H_2_S electrode was measured with the Analysenmesstechnik GmbH Amperometric H_2_S Micro-sensor. The buffer was 1 M BIS-TRIS titrated to pH 6.7 with HCl. In each experiment 70 mL of this buffer was used. A baseline of the concentration of H_2_S was first measured and then enough solid GYY-4137 to yield concentrations of 0.12 and 0.50 M were added. The aqueous GYY-4137 was capped with a rubber stopper that had holes to fit the sensors of the electrode. The H_2_S electrode was inserted through the holes and the cap was sealed tightly with parafilm.

### Statistical analysis

Statistical analysis was performed using IBM SPSS Statistics 25. Levene statistical analysis was performed to ensure homogeneity of variances. If the variances were homogenous then one-way ANOVA tests were used to show statistical relevance. Post-hoc test used to show statistical relevance with the control was the Dunnett’s multiple comparison test. If the variances were found to not be homogenous then the non-parametric Kruskal-Wallis test was performed. Data represented are mean±standard error with ** indicating α<0.05 and * indicating α<0.1.

## Results

### Hydrolysis of GYY-4137

**Fig 2:**
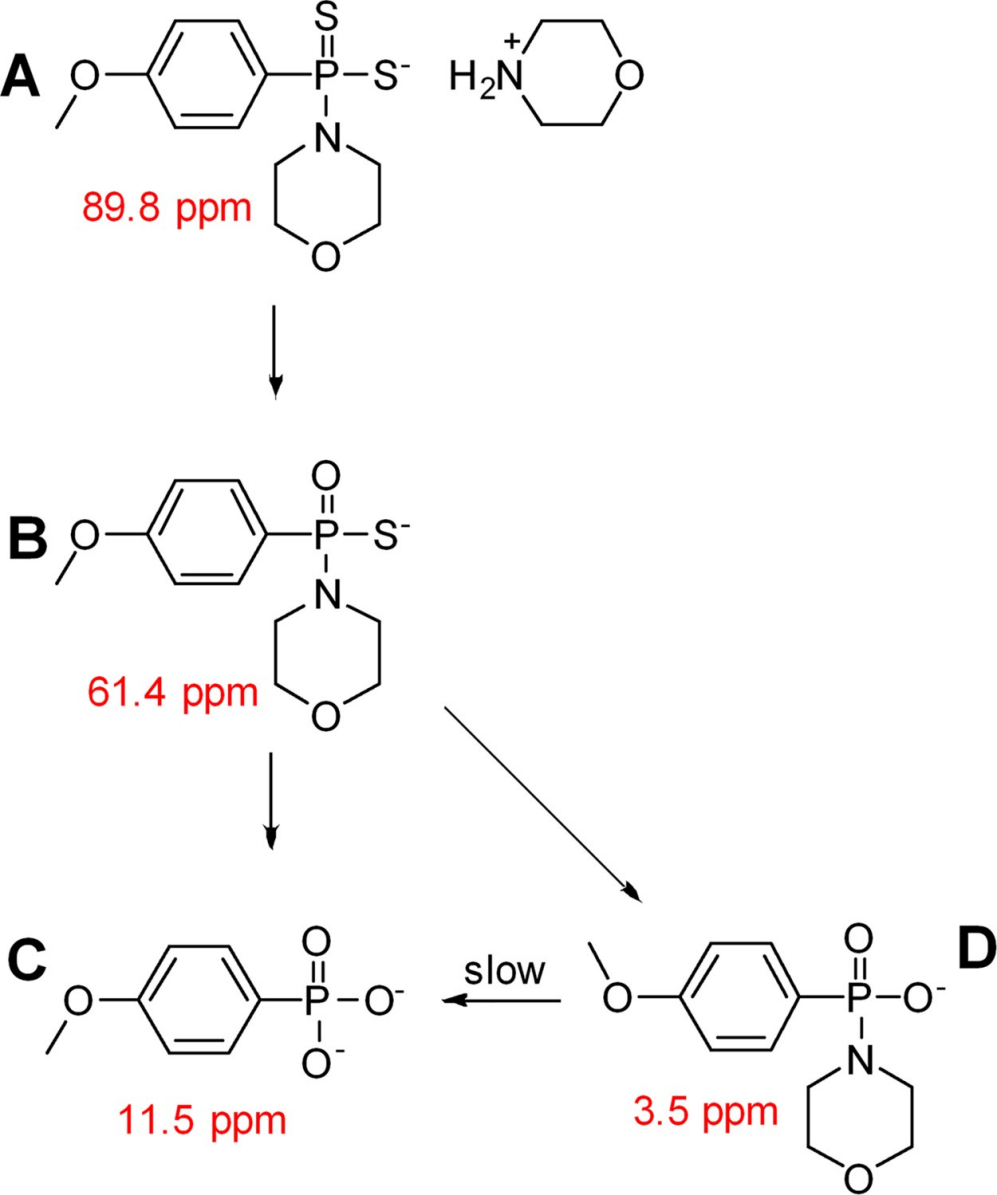
The hydrolysis of GYY-4137 in DMSO-d_6_ with residual H_2_O followed this reaction scheme. The numbers in red are the chemical shifts of the phosphorous peaks in the ^31^P NMR spectra.

The hydrolysis of GYY-4137 was followed in DMSO-d_6_ using ^31^P NMR spectroscopy for 109 days (Fig 2, S1 Fig and S2 Fig). Hydrolysis reactions require the presence of water as a reagent, and this reaction used the residual water in DMSO-d_6_ to complete this reaction. The peak due to GYY-4137 appeared at 89.8 ppm and lost 74% of its intensity after 7 days and was completely gone after 15 days due to hydrolysis with residual water in the DMSO-d_6_. The first product, **B**, of hydrolysis of GYY-4137 had a small peak at 61.4 ppm; its structure was identified in prior work.[44] Chemical **B** hydrolyzed to release morpholine and H_2_S and yielded the fully hydrolyzed chemical **C** at 11.5 ppm or it hydrolyzed to release H_2_S and yielded chemical **D** at 3.5 ppm. The identity of chemical **C** was shown in prior work, but chemical **D** has not been reported before.[44] Chemicals **C** and **D** were the only chemicals seen in the ^31^P NMR spectra from after day 15 to day 109, and the ratio of the integration of **C** to **D** remained constant at approximately 2.3.

We investigated the identity of chemical **D** by several methods. A tenfold excess of morpholine was added to a mixture of chemicals **C** and **D** to see if their equilibrium could be affected by excess morpholine, but no change in the ratio of chemicals was observed after 5 days (S3 Fig). A similar experiment was attempted by the addition of 10 molar equivalents of H_2_O to change the equilibrium but the ratio of **C** to **D** remained unchanged after 5 days (S4 Fig). Next, we hydrolyzed a new batch of GYY-4137 and after 20 days in DMSO-d_6_ only peaks **C** and **D** were observed at a ratio of 2.3 to 1. The NMR tube was heated in an 85°C oil bath for 24 hours and chemical **D** completely disappeared and the only peak in the NMR spectrum was due to chemical **C** (S5 Fig). Attempts to isolate chemical **D** were unsuccessful.

The hydrolysis of GYY-4137 was also monitored in water by ^31^P NMR spectroscopy at concentrations above and below its solubility limit of 0.13 M. At room temperature less than 3% of GYY-4137 hydrolyzed in water at concentrations of 0.5 M and 0.12 M over 35 days (S6 Fig and S7 Fig).

**Fig 3:**
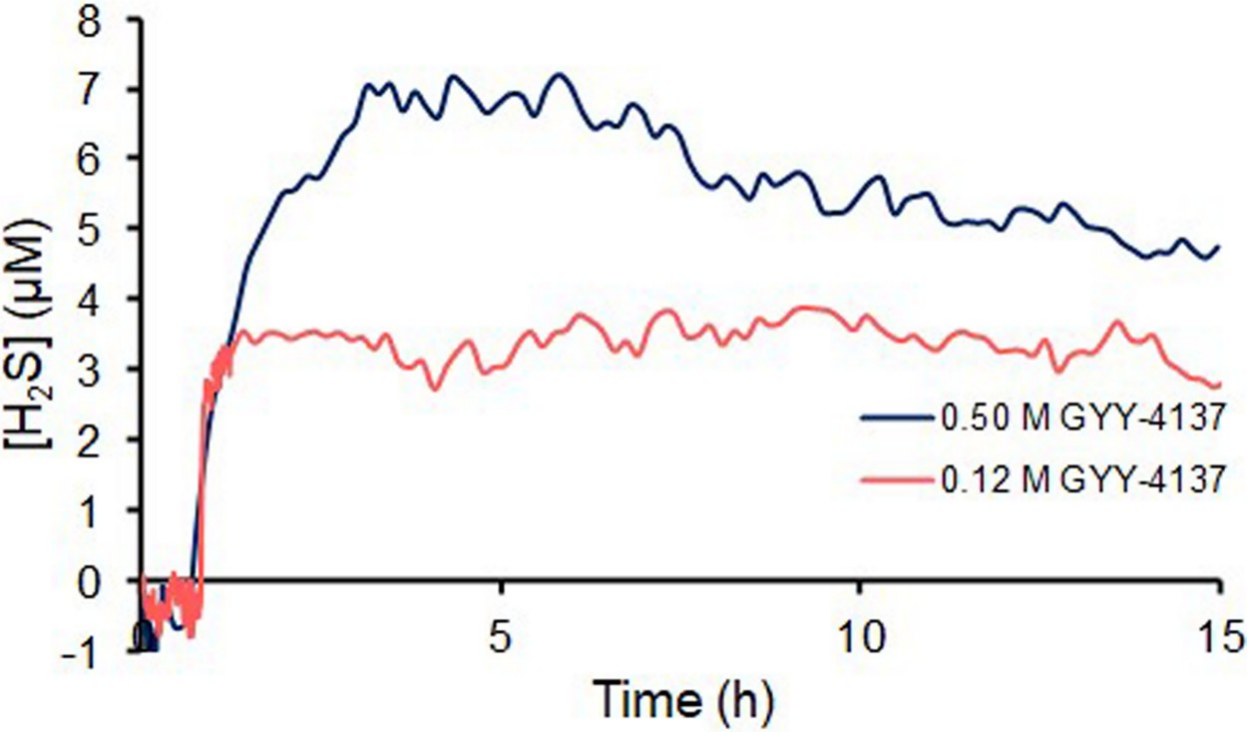
The concentration of H_2_S in water after addition of GYY-4137. The GYY-4137 was added to the aqueous buffer after 30 min.

The hydrolysis of GYY-4137 was also investigated using a H_2_S electrode that continuously measured the concentration of H_2_S (Fig 3). These experiments were completed at a pH of 6.7 to approximate the pH of soil in Iowa. Aqueous GYY-4137 at concentrations of 0.5 M and 0.12 M were investigated using the H_2_S electrode. These concentrations were chosen to investigate the hydrolysis of GYY-4137 at a concentration close to its solubility limit and approximately 4x its solubility limit. A baseline of the concentration of H_2_S was first measured in the absence of GYY-4137 and then GYY-4137 was added to the vessel. The concentration of H_2_S was approximately 5 and 3 μM after 15 h for the concentrations of 0.5 M and 0.12 M GYY-4137 respectively.

**Fig 4:**
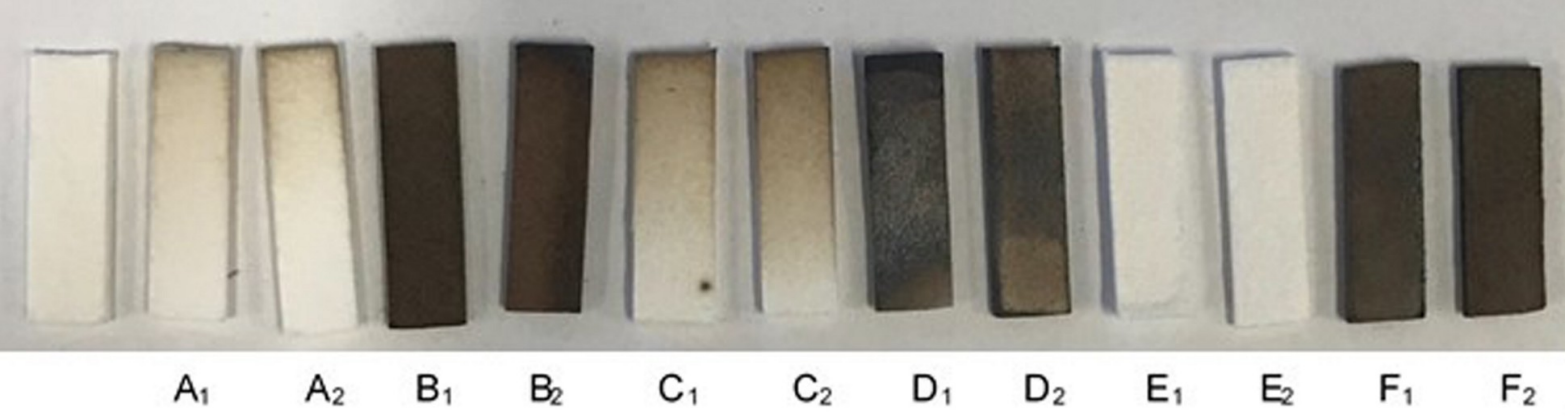
Lead strips were placed in capped vials with aqueous GYY-4137 or NaSH but were not allowed to contact the aqueous solutions. The strip on the left was not exposed to GYY-4137 or NaSH. Samples A_1_, A_2_, C_1_, C_2_, E_1_, and E_2_ were exposed to aqueous GYY-4137 and samples B_1_, B_2_, D_1_, D_2_, F_1_, and F_2_ were exposed to NaSH. Samples C_1_, C_2_, D_1_, and D_2_ had potting mix used to grow plants mixed with aqueous GYY-4137 or NaSH. Samples E_1_, E_2_, F_1_, and F_2_ used Iowa topsoil mixed with aqueous GYY-4137 or NaSH.

The effect of soil on the degradation of GYY-4137 was investigated using lead acetate strips that turn from white to black when exposed to H_2_S. An example of a lead strip not exposed to H_2_S is shown on the far left of Fig 4. In all of the experiments the lead strips were sealed in a vial with 0.6 mL (subscript 1) or 1.2 mL (subscript 2) aqueous GYY-4137 or NaSH but were not allowed to come into contact with the water. H_2_S_(aq)_ released from GYY-4137 was in equilibrium with H_2_S_(g)_ and was not allowed to escape from the capped vial. The H_2_S_(g)_ diffused to the lead acetate strips and reacted to produce highly insoluble, black PbS. Lead strips enclosed in vials with aqueous GYY-4137 at a concentration of 0.5 M for 10 days had a slight browning on their surface (A_1_ and A_2_) which is consistent with the release of a small amount of H_2_S. Two sets of experiments were completed where the potting mix used in this work was added to the aqueous GYY-4137 (C_1_ and C_2_) and topsoil from a nearby field was added to aqueous GYY-4137 (E_1_ and E_2_). Similar browning of the lead strips was observed for samples with and without soil used in this work, but little to no browning was observed when Iowa topsoil was added to aqueous GYY-4137. Samples B_1_, B_2_, D_1_, D_2_, F_1_, and F_2_ were positive controls in which NaSH was added to the vials rather than GYY-4137 to demonstrate the dark colors of lead strips.

### Effect of GYY-4137 on radishes

**Fig 5:**
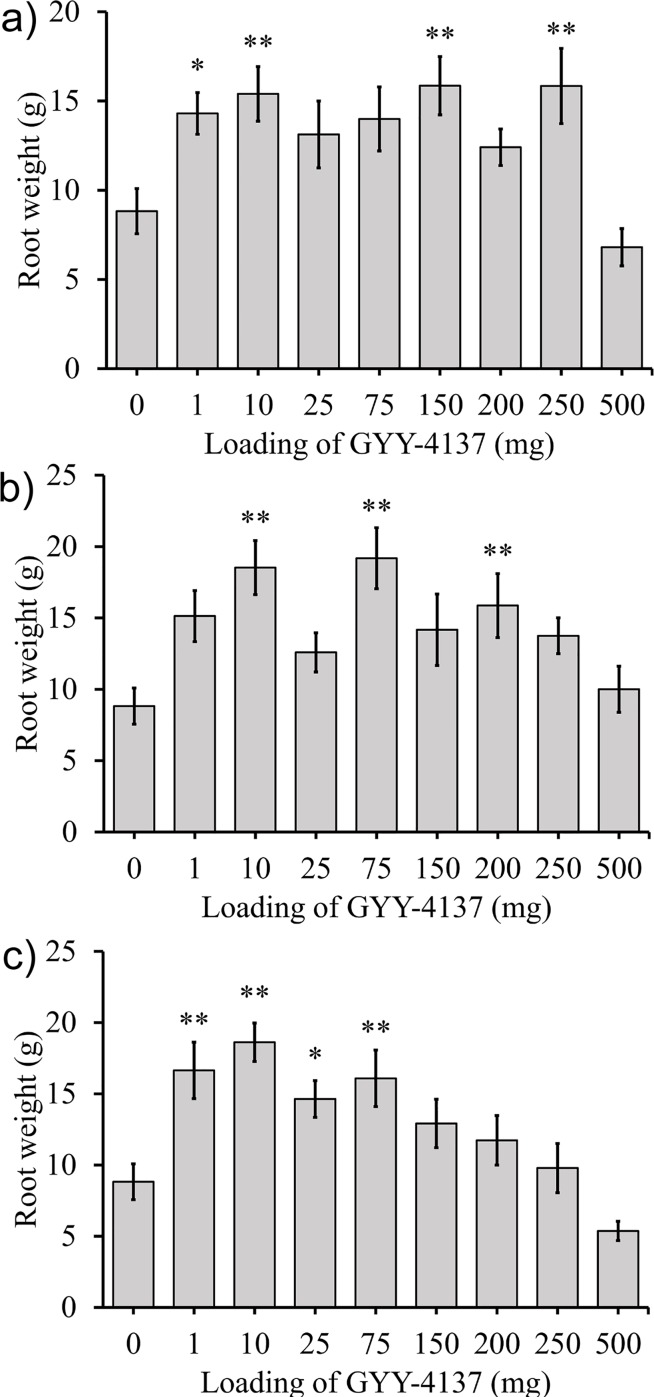
Average radish root weight of radish plants exposed to various applications and doses of GYY-4137 is shown. a) GYY-4137 was added directly to potting mix before planting. b) Aqueous GYY-4137 was added to the potting mix near seeds after they were planted. c) The plants were dosed weekly with different amounts of GYY-4137. Data represented are mean±standard error with ** indicating α<0.05 and * indicating α<0.1.

Radishes were grown using methods 1, 2, and 3 for delivering GYY-4137 for 40 days. Results for method 1 (blending GYY-4137 powder into the potting mix) is shown in Fig 5A. Fig 5B shows results from method 2 (adding GYY-4137 as an aqueous solution at the time of planting). Fig 5C shows the response to weekly addition of GYY-4137 solutions (method 3) at loadings of 0 to 500 mg. In all treatments, low levels of GYY-4137 increased root weight, and high loadings resulted in lower root weighs. 10 mg per plant consistently yielded increased root weight, regardless of delivery method.

### Effect of GYY-4137 on peas

**Fig 6:**
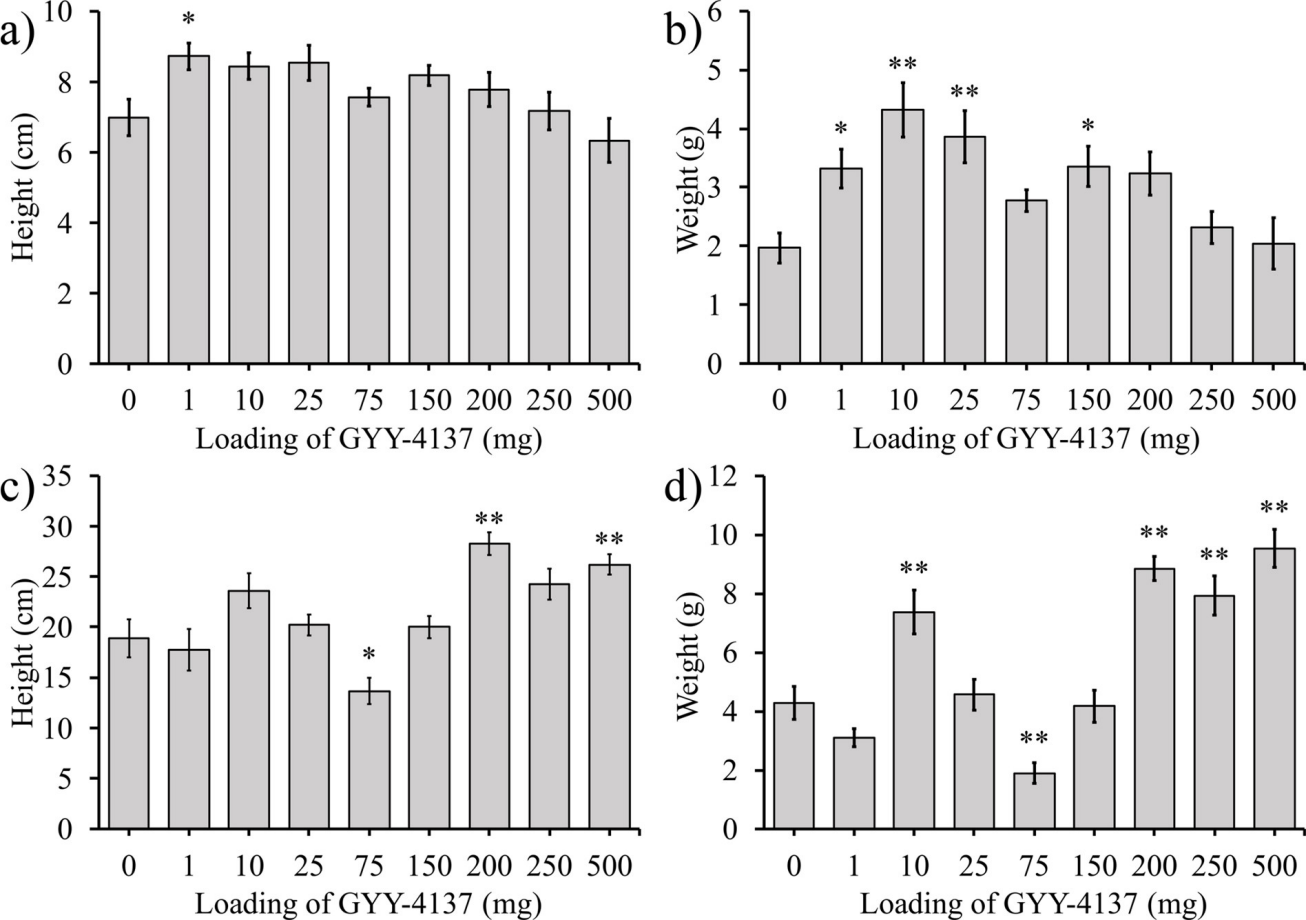
Effect of GYY-4137 on pea shoot height and fresh weight. The a) heights at 3 weeks, (b) weights at 3 weeks, c) heights at 6 weeks, and (d) weights at 6 weeks are shown. Data represented are mean±standard error with ** indicating α<0.05 and * indicating α<0.1.

Pea plants were grown for 3 and 6 weeks with different loadings of GYY-4137 provided by method 1 (Fig 6). At 3 weeks the height of the shoot to the node of the uppermost visible leaf was measured and the fresh weight was determined (Fig 6A and 6B). At 6 weeks the second set of plants were similarly analyzed (Fig 6C and 6D). After 3 weeks, the lowest loading gave the greatest increase in both height and weight. Interestingly, at 6 weeks the low loadings had no significant effect but higher loadings of 200–500 mg resulted in a roughly 50% increase in height and more than 100% increase in fresh weight.

**Fig 7:**
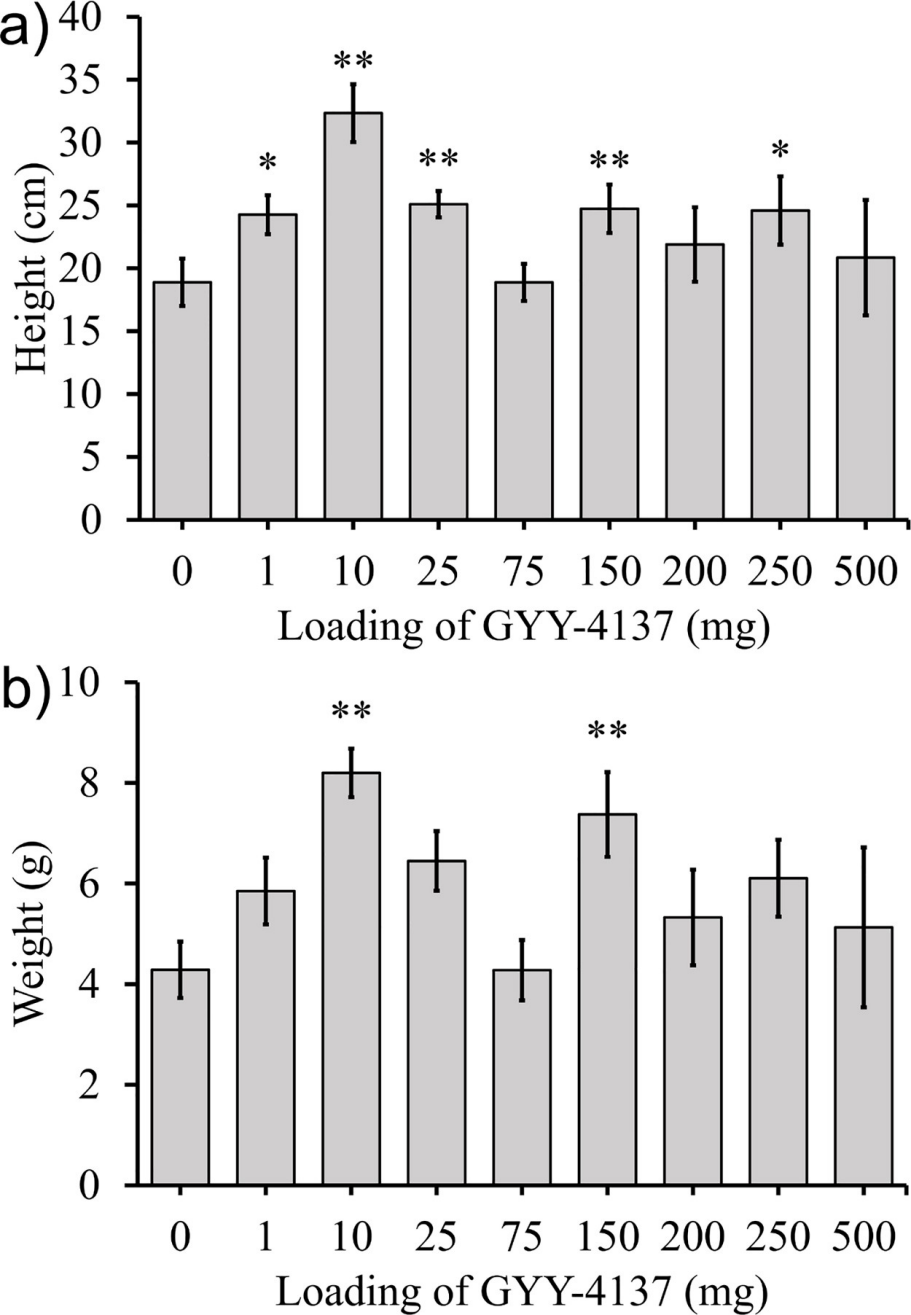
GYY-4137 was administered to pea plants by method 2. The a) height and b) fresh weight of pea shoots after 6 weeks is shown Data represented are mean±standard error with ** indicating α<0.05 and * indicating α<0.1.

**Fig 8:**
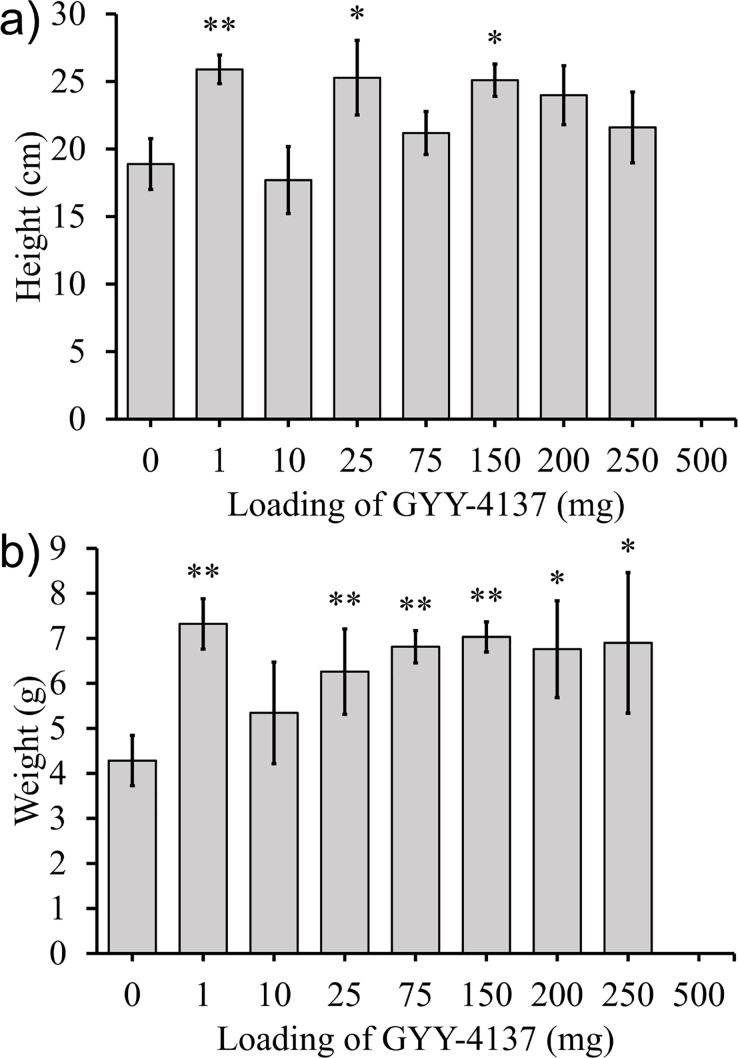
GYY-4137 was administered weekly to the pea plants by method 3. Shoot a) height and b) weight of pea shoots at 6 weeks is shown. Data represented are mean±standard error with ** indicating α<0.05 and * indicating α<0.1.

Two other sets of pea plants were grown using methods 2 and 3 to deliver GYY-4137. In one set of experiments, peas were grown for 6 weeks after method 2 delivery (a one-time application of aqueous GYY-4137). Shoot height and fresh weight was determined (Fig 7). A second set received GYY-4137 by method 3 (weekly application of GYY-4137 solutions). After 6 weeks the height and weight of the shoots were measured (Fig 8).

### Effect of GYY-4137 on lettuce plants

**Fig 9:**
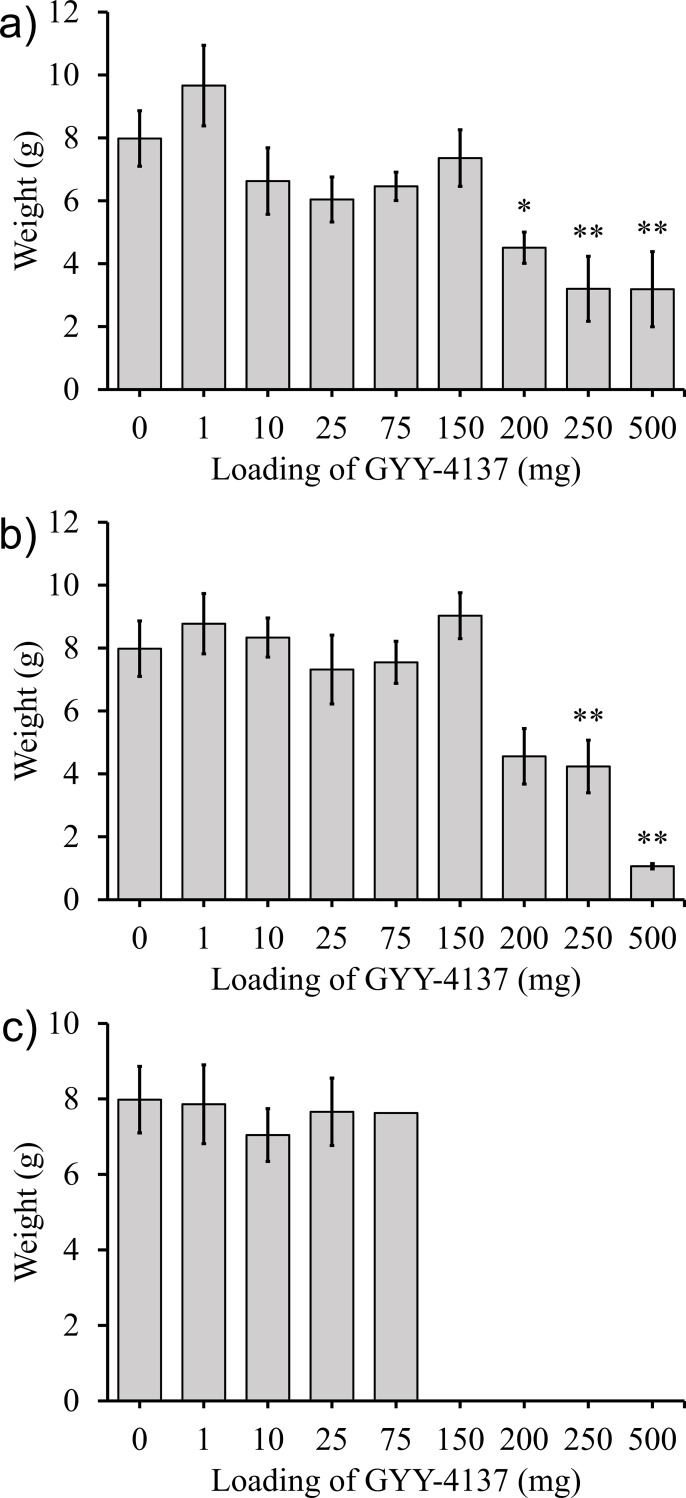
The average mass of the head of lettuce for lettuce plants with GYY-4137 applied by a) method 1, b) method 2, or c) method 3. In c) only one plant at 75 mg survived to harvest and no plants survived at higher loadings. Data represented are mean±standard error with ** indicating α<0.05 and * indicating α<0.1.

Four different experiments were completed with lettuce plants to investigate the effect of GYY-4137 on growth after 6 weeks, as assayed by shoot fresh weight. In two sets of experiments GYY-4137 was added to the potting mix prior to planting. In the first set, GYY-4137 was thoroughly mixed with the soil prior to the addition of seeds (method 1) (Fig 9A). In a second set of experiments, aqueous GYY-4137 was added directly adjacent to the seed at planting, rather than being uniformly distributed in the pot (Fig 9B). In a third set of experiments, GYY-4137 was added weekly to the soil above the roots (method 3) for 5 weeks (Fig 9C). In the last set of experiments, plants exposed to doses of GYY-4137 higher than 75 mg did not survive to the end of the 6 week period.

**Fig 10:**
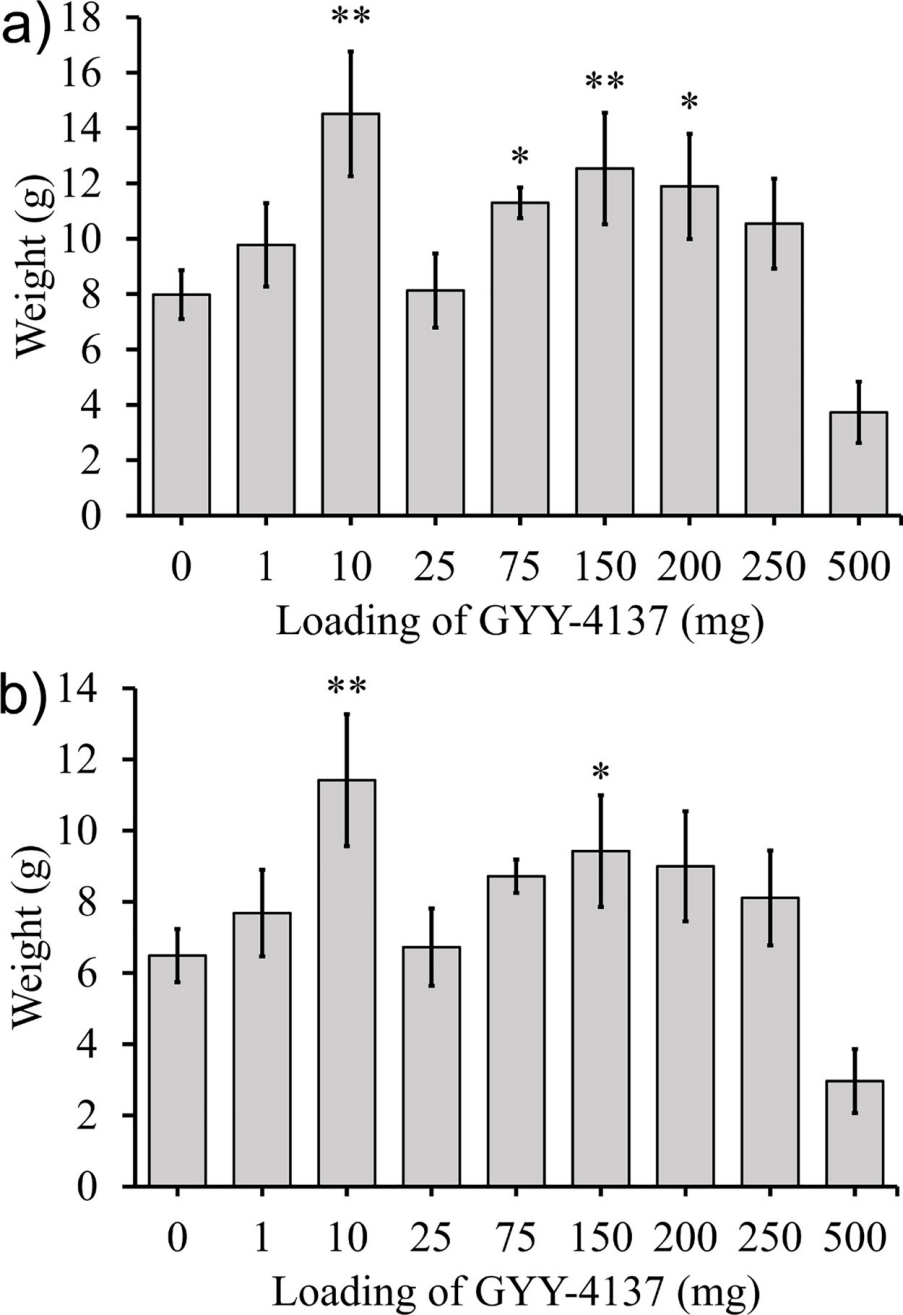
Lettuce plants were exposed to GYY-4137 at day 0 in the soil and at days 7, 14, 21, and 35 by dosing of aqueous GYY-4137 on top of the stem. The plants were grown for 6 weeks, and the a) average mass of the stem and leaves and b) average mass of leaves is shown. Data represented are mean±standard error with ** indicating α<0.05 and * indicating α<0.1.

In a fourth set of experiments GYY-4137 was applied by method 4 to lettuce seeds. It was added adjacent to the seeds at planting and at days 7, 14, 21, 28, and 35 aqueous solutions of GYY-4137 were applied to the leaves. Shoots were harvested and weighed at week 6 (Fig 10).

## Discussion

### Release of H_2_S from GYY-4137

Although it is regularly reported that GYY-4137 hydrolyzes to release H_2_S in water, its rates of hydrolysis in water and other solvents is uneven. In organic solvents it hydrolyzes with residual water over days to weeks. In experiments reported here, it hydrolyzed by 74% in 7 days in DMSO-d_6_ and in another paper it hydrolyzed by 50% in 13 days when dissolved in chloroform or acetone.[44] In contrast, its hydrolysis in water was very sluggish: less than 3% hydrolyzed after 35 days as measured by ^31^P NMR spectroscopy. Similar results were found when the release of H_2_S from GYY-4137 was monitored with an H_2_S electrode. At concentrations of 0.12 M and 0.50 M at a pH of 6.7 the steady state concentration of H_2_S was approximately 3 and 5 μM which were approximately 10^5^ lower concentrations than GYY-4137. The concentration of H_2_S did not scale linearly with the concentration of GYY-4137 because the solubility limit of GYY-4137 was only 0.13 M. At a concentration of 0.50 M GYY-4137 much of the GYY-4137 was an insoluble solid at the bottom of the vial. These experiments demonstrate that GYY-4137 that is swollen in water, but not dissolved, will release low levels of H_2_S. These results demonstrate that the local solvent environment for the hydrolysis of GYY-4137 is important for consideration of its rate of degradation. The presence of organic chemicals near GYY-4137 can strongly influence the rate of release of H_2_S. Furthermore, GYY-4137 that is not fully dissolved in H_2_O will also release H_2_S which is an important consideration for its applications in agriculture when GYY-4137 is added to soil for extended periods of time.

The release of H_2_S from GYY-4137 in the presence of two different growth media was investigated. In a bark-based potting mix used here, H_2_S was detected by darkening of a lead acetate strip after 10 days. When Iowa topsoil dug locally was used, no release of H_2_S from GYY-4137 was observed using lead acetate strips. Although qualitative, these results further demonstrate the importance of considering the local environment of GYY-4137.

These experiments describe the importance of considering the soil composition as well as concentrations of aqueous GYY-4137 in measurements of plant growth responses. Water from dilute aqueous solutions of GYY-4137 added to soil may evaporate leaving behind solid GYY-4137 that releases H_2_S when watered or concentrated aqueous solutions of GYY-4137 that slowly release H_2_S. The loading of organic material in the soil may affect the release of H_2_S from GYY-4137 by providing a nonpolar, organic environment that will accelerate the release of H_2_S. It is also clear that it is not necessary to have enzymes to release H_2_S from GYY-4137, although the presence of enzymes may speed the release of H_2_S.

### Effect of GYY-4137 on the harvest yield of radishes

Although prior work from the 1990s using exogenous H_2_S on radishes demonstrated reduced growth rates, our experiments demonstrated that GYY-4137 applied on radish plants could increase their root harvest weight (Fig 5).[68–70] Radishes exposed to GYY-4137 increased in weight by up to 117% compared to radishes grown in the absence of GYY-4137. In all three sets of experiments several loadings of GYY-4137 produced statistically significant increases in the weight of the radish roots. Loadings as low as 1 mg and as high as 250 mg of GYY-4137 gave statistically significant increases in the weight of the radish roots. This result is important because it demonstrates that the beneficial effect of GYY-4137 occurred over a large range of doses administered in different locations (i.e. distributed throughout the soil or added near the seed). It is also notable that the addition of only 1 mg of GYY-4137 led to a statistically significant increase in the weight of the radish roots because the hydrolysis of 1 mg of GYY-4137 will result in the release of only 0.18 mg of H_2_S in total over a prolonged period.

A possible explanation for the difference between this work and prior publications is that in prior work the concentrations of aqueous H_2_S applied to radish plants ranged from 1.56 to 3.12 mM. Although much of the H_2_S evaporated due to its low boiling point of -60°C, these concentrations may have been too high. In contrast, this work used much lower doses of GYY-4137 which released significantly lower doses of H_2_S over several weeks.

### Effect of GYY-4137 on the growth of pea plants

A comparison of the effect of different loadings of GYY-4137 on pea plants at 3 and 6 weeks shows an important effect not previously reported. The plants harvested at 3 weeks had the largest heights and weights at 1, 10, and 25 mg of GYY-4137 that was thoroughly mixed in the soil (Fig 6A and 6B). All loadings of GYY-4137 from 1 to 250 mg increased height and weight of the pea shoots, but the statistically significant effects were observed only at the lower loadings of GYY-4137. In fact, at 10 mg of GYY-4137 pea plants doubled in weight compared to the control plants grown in the absence of GYY-4137. Pea plants harvested after 6 weeks of growth had the greatest shoot lengths and weights with the 200, 250, and 500 mg loadings of GYY-4137 in the soil (Fig 6C and 6D). The weights of pea plants at loadings of 200, 250, and 500 mg nearly or more than doubled in weight compared to the control plants grown in the absence of GYY-4137. Except for the weight of pea plants at 10 and 75 mg of GYY-4137, pea plants harvested after 6 weeks at loadings from 1–150 mg were statistically the same as the control plants.

No prior publications have reported the shifting of the effect of GYY-4137 or aqueous H_2_S on the growth of plants as a function of time. Prior work with exogenous H_2_S has focused on the first week of growth post germination of plants or their survival to environmental stressors and have not investigated the change in growth of plants at different loadings of GYY-4137 at different time periods. No prior work has demonstrated that the optimal loading of H_2_S or GYY-4137 may change at different periods in the lifecycle of plants. These results demonstrate the importance of considering how H_2_S can affect the growth of plants over their entire lifecycles.

Pea plants grown for 6 weeks where an initial dose of GYY-4137 was added adjacent to the seeds (Fig 7) had a different response to seeds planted with GYY-4137 mixed throughout the soil (Fig 6C and 6D). The addition of GYY-4137 close to the seeds resulted in statistically significant increases in shoot length and weight of the pea plants at a wide range of loadings of 1 to 250 mg of GYY-4137. A comparison between Fig 6C and 6D with Fig 7 demonstrate the importance of considering where GYY-4137 is added relative to the seeds. Since GYY-4137 releases H_2_S that can escape as a gas, it is more likely that GYY-4137 administered near the seeds will release H_2_S that the plants can capture.

GYY-4137 and H_2_S are well-known to be toxic to plants and this effect was observed when GYY-4137 was administered to the plants every week (Fig 8).[50, 63, 71, 72] Plants exposed to 500 mg of GYY-4137 germinated, but they perished before they could be harvested at 6 weeks. Similarly, only 42% and 50% of the plants exposed to weekly doses of 250 and 200 mg respectively of GYY-4137 survived to harvest at 6 weeks. This figure also demonstrates that pea plants exposed to 1 to 250 mg of GYY-4137 every week had statistically significantly higher weights than control plants not exposed to GYY-4137. The graphs in Fig 8 demonstrate that weekly dosings of GYY-4137 may be beneficial to plants but that at high loadings it will decrease their survival.

### Effect of GYY-4137 on the growth of lettuce plants

Three experiments with GYY-4137 added to soil to lettuce seeds led to poor growth and low survival of the plants. When GYY-4137 was thoroughly mixed into the soil in a pot at day 0 and the plants were regularly watered for 6 weeks, the weight of the heads of lettuce exposed to GYY-4137 were not statistically greater than the control (Fig 9A). At loadings of 200, 250, and 500 mg of GYY-4137 the heads of lettuce had statistically significantly reduced masses compared to the control plants. Experiments when GYY-4137 was added to the soil adjacent to the seeds also did not show a positive effect on the weight of the heads of lettuce. At 200 to 500 mg loadings of GYY-4137 only 33% of the seeds germinated which provided further evidence that GYY-4137 near the seeds did not have a positive effect on the growth of lettuce plants (Fig 9B). When GYY-4137 was added to the soil every week, the plants had worse survival rates and weights at harvest (Fig 9C). The control plants and plants exposed to 1 mg of GYY-4137 every week had 92% survival to week 6, but the plants exposed to 10 mg per week had only a 58% survival to week 6. None of the plants exposed to weekly doses of 150 mg and higher of GYY-4137 survived to week 6.

A fourth set of experiments where GYY-4137 was added to the soil at day 0 and to the leaves at days 7, 14, 21, 28, and 35 had sharply different results (Fig 10). These were the only experiments to add GYY-4137 directly to the leaves, in the other experiments GYY-4137 was added to the soil. Statistically different weights of the stem and leaves at 10, 75, 150, and 250 mg of GYY-4137 were observed. The effect was largest for the plants grown with exposure to 10 mg of GYY-4137 per week which had an increase in weight of lettuce stem and leaves by 83% compared to the control plants not exposed to GYY-4137.

The increased growth of lettuce plants exposed to GYY-4137 on the leaves was consistent with the results from 1978 and 1979 where lettuce plants were grown in greenhouses continuously exposed to gaseous H_2_S for the length of the growing season (2 to 3 months depending on the time of the year of the growing season).[61, 62] The results in this paper demonstrated that the location of administration of GYY-4137 is an important parameter to consider. When GYY-4137 was applied to the soil its effect was neutral or negative on the growth of lettuce plants, but application of GYY-4137 to the leaves had a strong positive effect on the growth of the plants.

## Conclusion

The hydrolysis of GYY-4137 is complex and proceeds significantly faster in organic solvents than in water. Less than 3% of GYY-4137 hydrolyzed in water after 5 weeks, but in DMSO-d_6_ GYY-4137 was over 74% hydrolyzed within 7 days. This demonstrated the importance of considering the local environment of GYY-4137 when used in agricultural studies and also further demonstrated that GYY-4137 does release H_2_S by hydrolysis in water. The rate of release of H_2_S from GYY-4137 is not straightforward and may involve its uptake into plants through the roots or leaves.

This research demonstrated the dual importance of the location and duration of application of GYY-4137. Prior work with lettuce exposed to gaseous H_2_S exhibited increased growth but when GYY-4137 was added to the pots of lettuce plants only negative effects were observed. In contrast, when GYY-4137 was applied to lettuce leaves the weight of the leaves increased by up to 82%. The location of application of GYY-4137 was clearly an important aspect of how it affected lettuce plant growth and should be considered in future work with other plants. The importance of the duration of release of H_2_S was shown by the different results for pea plants harvested at 3 and 6 weeks. The loading of GYY-4137 that had the most positive effect shifted to higher doses for plants harvested at 6 weeks compared to 3 weeks. This result was unexpected and unreported. The origin for the change in effect of plants to different doses of GYY-4137 at different times is important to consider and should be studied in future work.

This work demonstrated that H_2_S has the potential to increase the harvest yields of radishes and lettuce. Importantly, only milligram quantities of H_2_S were needed to have a large effect on the growth of these crops, but the location and number of times GYY-4137 needed to be applied differed for the crops. Although this work was completed in a greenhouse, it may have implications for the growth of crops in agricultural fields.

## Supporting information

S1 Fig The degradation pathway of GYY-4137 in DMSO-d_6_ is shown. The ^31^P peaks for each chemical is shown to illustrate its splitting pattern. No internal standard was used to collect the ^31^P NMR spectra. The usual internal standard is phosphoric acid, but this was not used due to concerns that it would affect the degradation rate of the GYY-4137. The lack of an internal standard resulted in a small drift for the ^31^P peaks in the spectra shown below. To ensure that the drift of the NMR spectrometer was small, the chemical shift of phosophoric acid in a separate NMR tube was regularly checked and used to calibrate the instrument.
(TIF)

S2 Fig The ^31^P NMR spectra of the degradation of GYY-4137 in DMSO-d_6_ is shown at various time points. No change in the spectra was observed from day 22 to day 104.
(TIF)

S3 Fig The addition of water to a mixture of chemicals C and D did not change their relative amounts. At day 104 water (10 molar equivalents) was added to the NMR tube. At day 109 no change in ratio of chemicals **C** and **D** was observed by ^31^P NMR spectroscopy.
(TIF)

S4 Fig The addition of morpholine did not change the ratio of chemicals C and D. On day 109 morpholine (10 molar equivalents) was added to the NMR tube and no change of the integration of the peaks was observed on day 114 by ^31^P NMR spectroscopy.
(TIF)

S5 Fig Chemical D could convert to chemical C. GYY-4137 was heated in an NMR tube for 20 days to yield a mixture of chemicals **C** and **D**. The NMR tube was heated in 85°C oil bath for 24 hours. The ^31^P NMR spectrum on day 21 showed complete consumption of **D** leaving only compound **C**. This result indicated that **D** could convert to **C** but that the reaction was slow at room temperature.
(TIF)

S6 Fig GYY-4137 did not show any hydrolysis products in water by ^31^P NMR spectroscopy. GYY-4137 was completely dissolved at a concentration of 0.12 M in 90% H_2_O/D_2_O. The ^31^P NMR spectrum after 35 days showed less than 3% of the GYY-4137 hydrolyzed.
(TIF)

S7 Fig At a concentration of 0.50M GYY-4137 some hydrolysis was observed after 35 days in water. GYY-4137 was added to 90% H_2_O/D_2_O at a concentration of 0.50M. The solubility limit of GYY-4137 in water is approximately 0.13M so much of the GYY-4137 was a solid at the bottom of the NMR tube. The ^31^P NMR spectrum after 35 days showed less than 3% of the GYY-4137 hydrolyzed.
(TIF)
