# Supplementary figures and images for: Improved growth of pea, lettuce, and radish plants using the slow release of hydrogen sulfide from GYY-4137
Source: PLoS One. 2018 Dec 17;13(12):e0208732. doi: 10.1371/journal.pone.0208732 (PMC6296661; doi:10.1371/journal.pone.0208732)

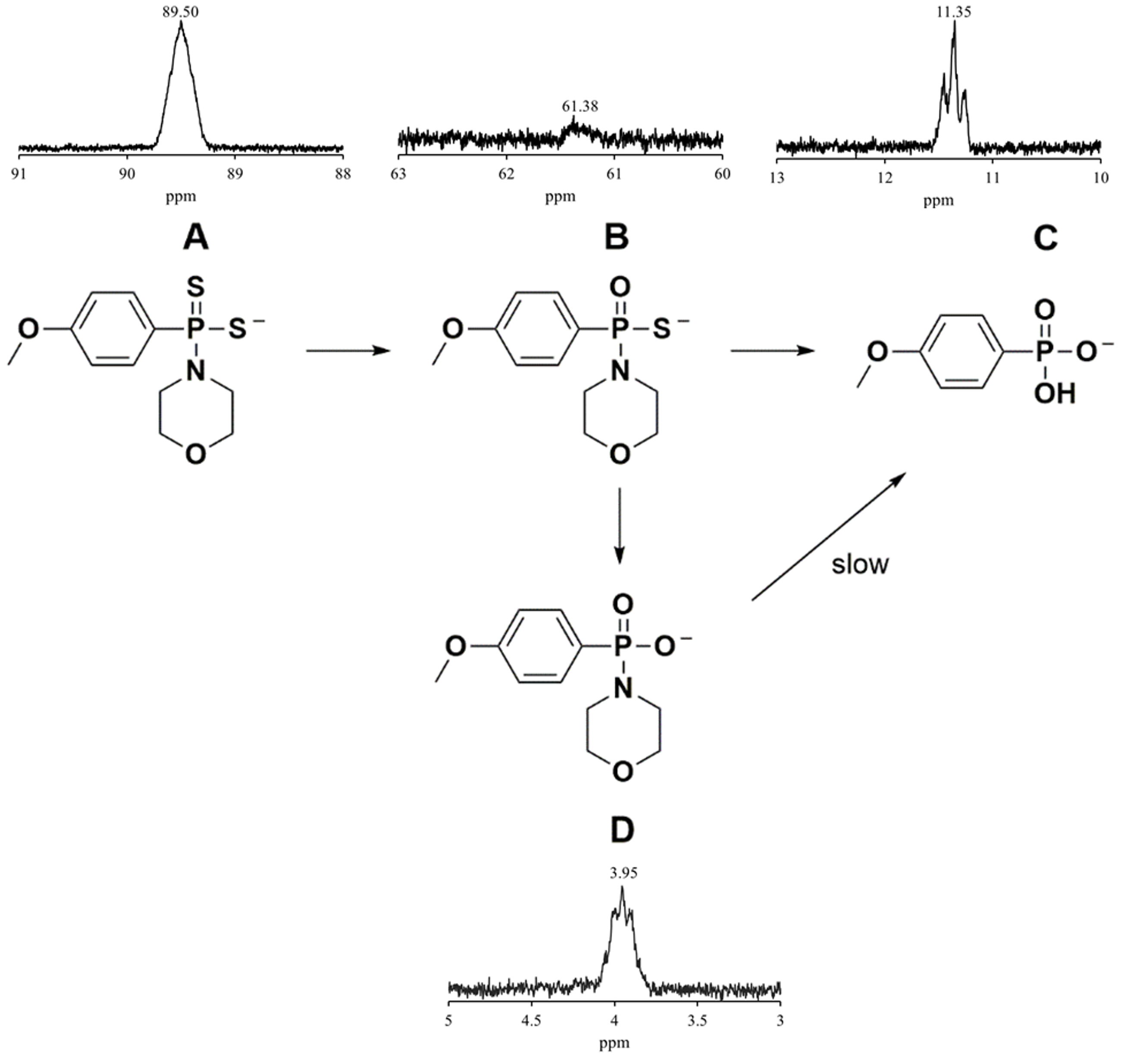

Supplement: S1 Fig — The 31P peaks for each chemical is shown to illustrate its splitting pattern. No internal standard was used to collect the 31P NMR spectra. The usual internal standard is phosphoric acid, but this was not used due to concerns that it would affect the degradation rate of the GYY-4137. The lack of an internal standard resulted in a small drift for the 31P peaks in the spectra shown below. To ensure that the drift of the NMR spectrometer was small, the chemical shift of phosophoric acid in a separate NMR tube was regularly checked and used to calibrate the instrument. (TIF) [file pone.0208732.s001.tif]

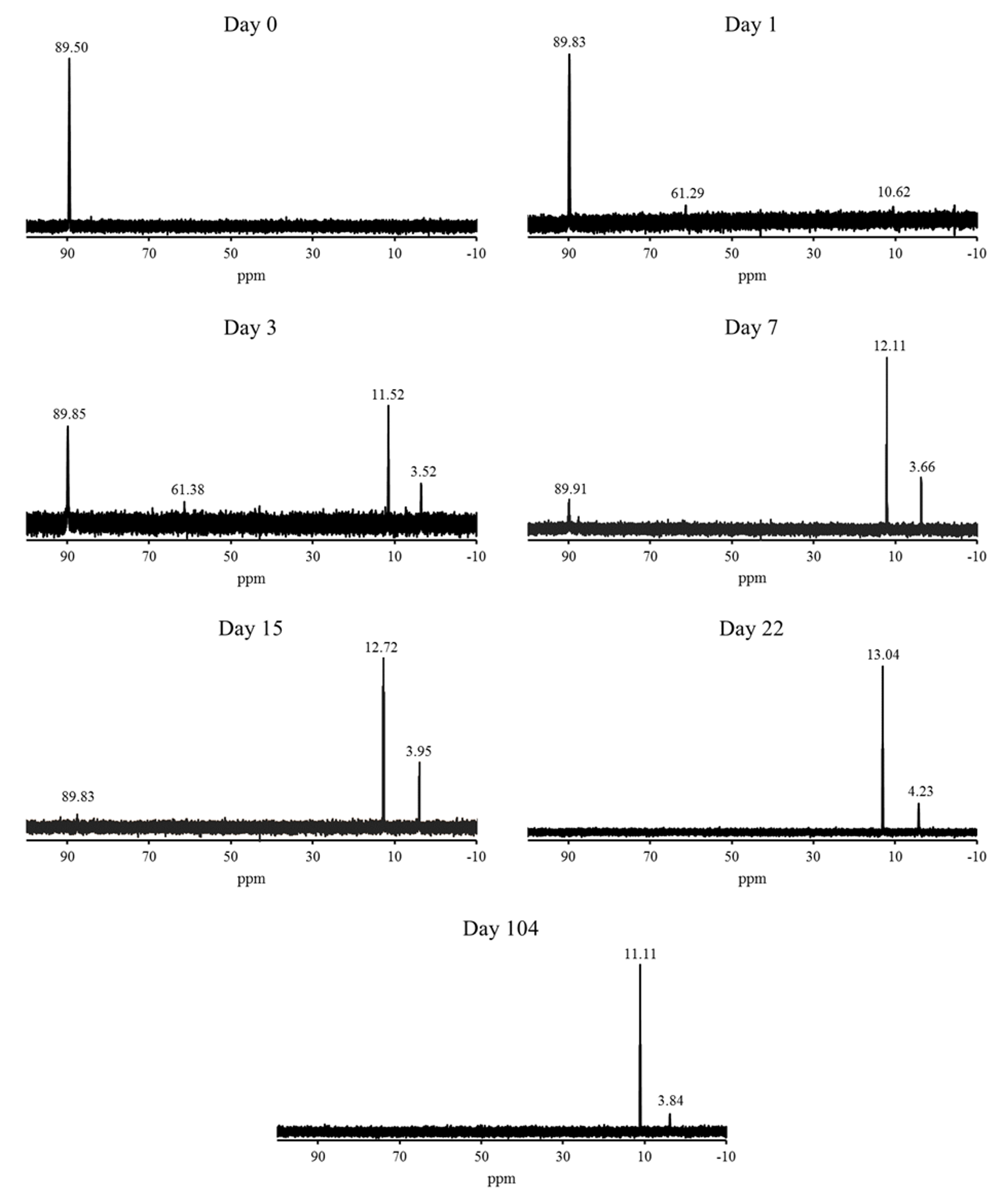

Supplement: S2 Fig — No change in the spectra was observed from day 22 to day 104. (TIF) [file pone.0208732.s002.tif]

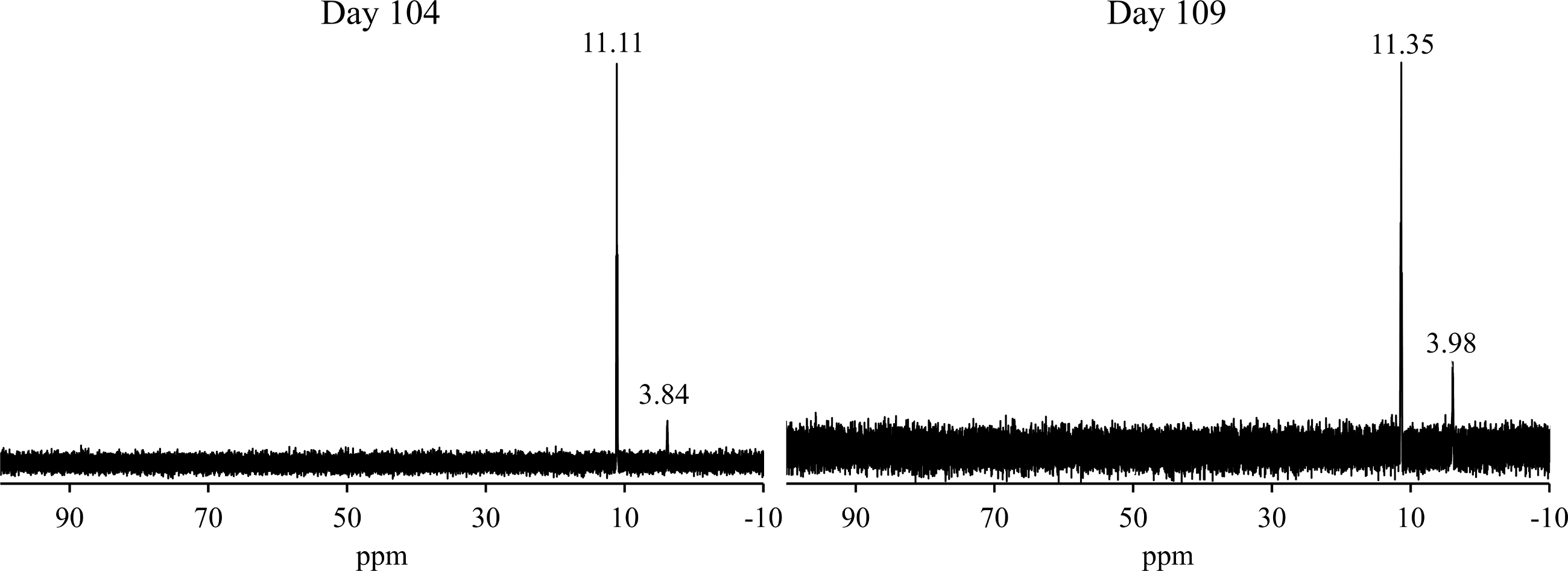

Supplement: S3 Fig — At day 104 water (10 molar equivalents) was added to the NMR tube. At day 109 no change in ratio of chemicals C and D was observed by 31P NMR spectroscopy. (TIF) [file pone.0208732.s003.tif]

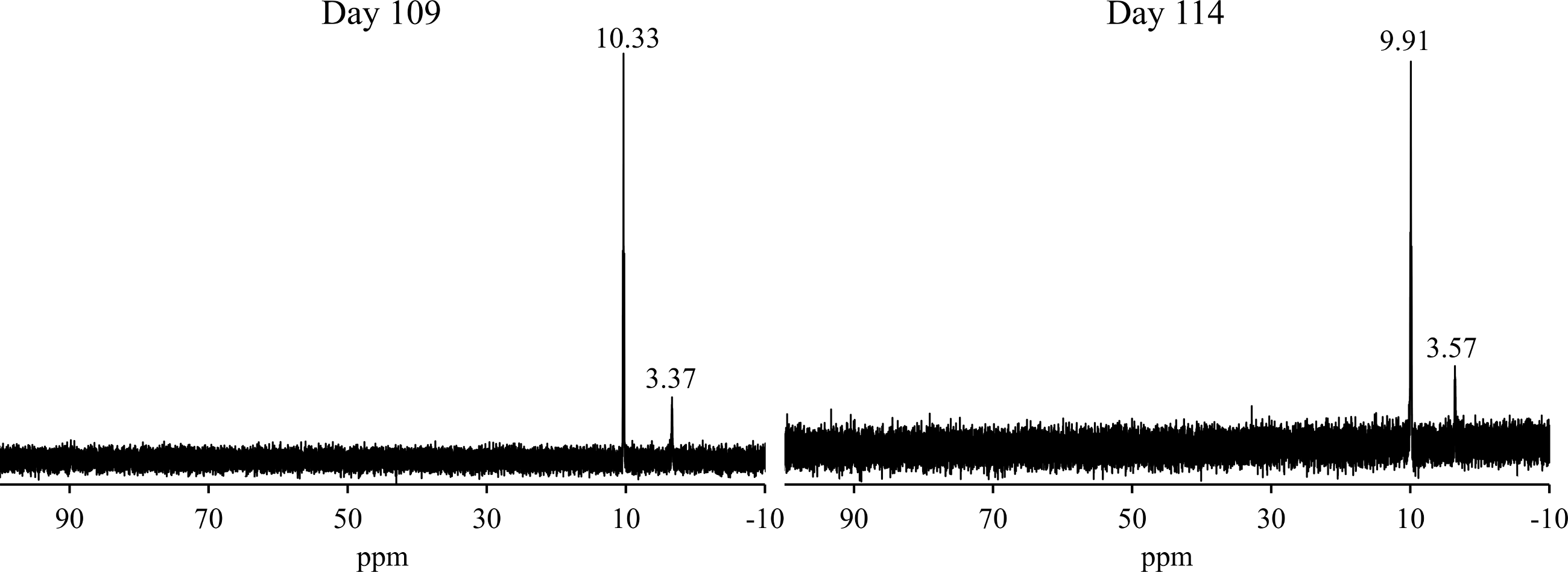

Supplement: S4 Fig — On day 109 morpholine (10 molar equivalents) was added to the NMR tube and no change of the integration of the peaks was observed on day 114 by 31P NMR spectroscopy. (TIF) [file pone.0208732.s004.tif]

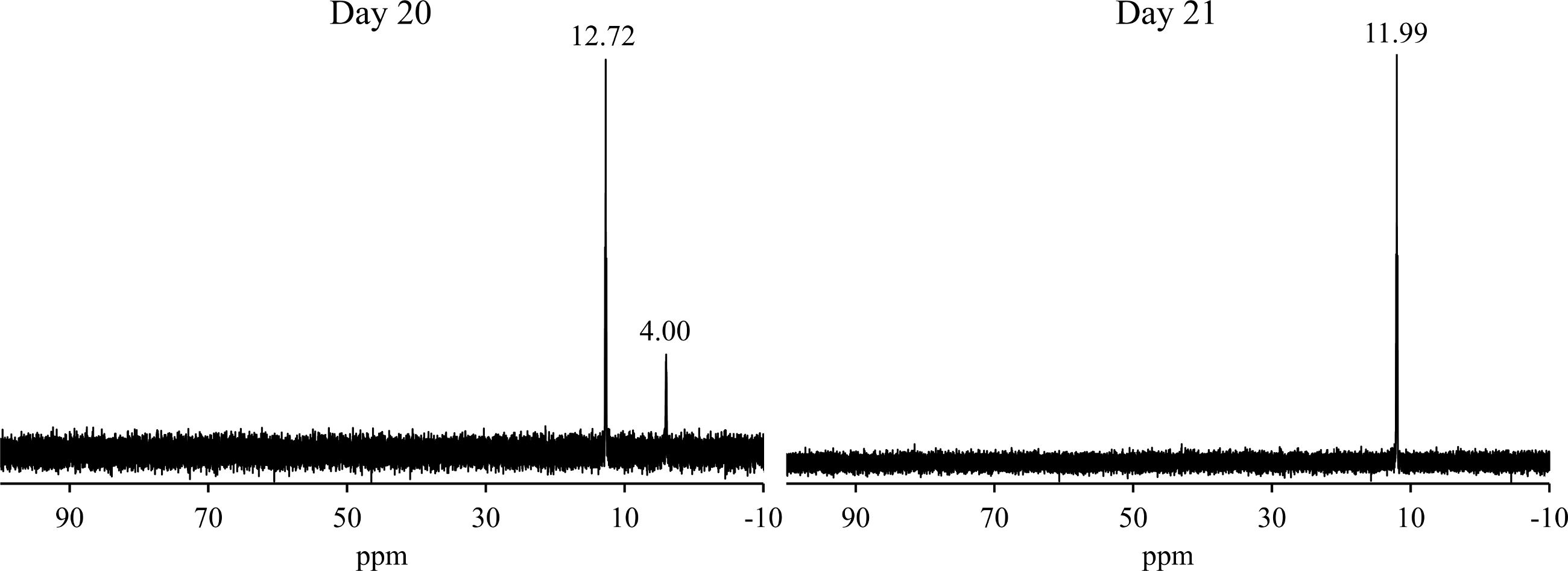

Supplement: S5 Fig — GYY-4137 was heated in an NMR tube for 20 days to yield a mixture of chemicals C and D. The NMR tube was heated in 85°C oil bath for 24 hours. The 31P NMR spectrum on day 21 showed complete consumption of D leaving only compound C. This result indicated that D could convert to C but that the reaction was slow at room temperature. (TIF) [file pone.0208732.s005.tif]

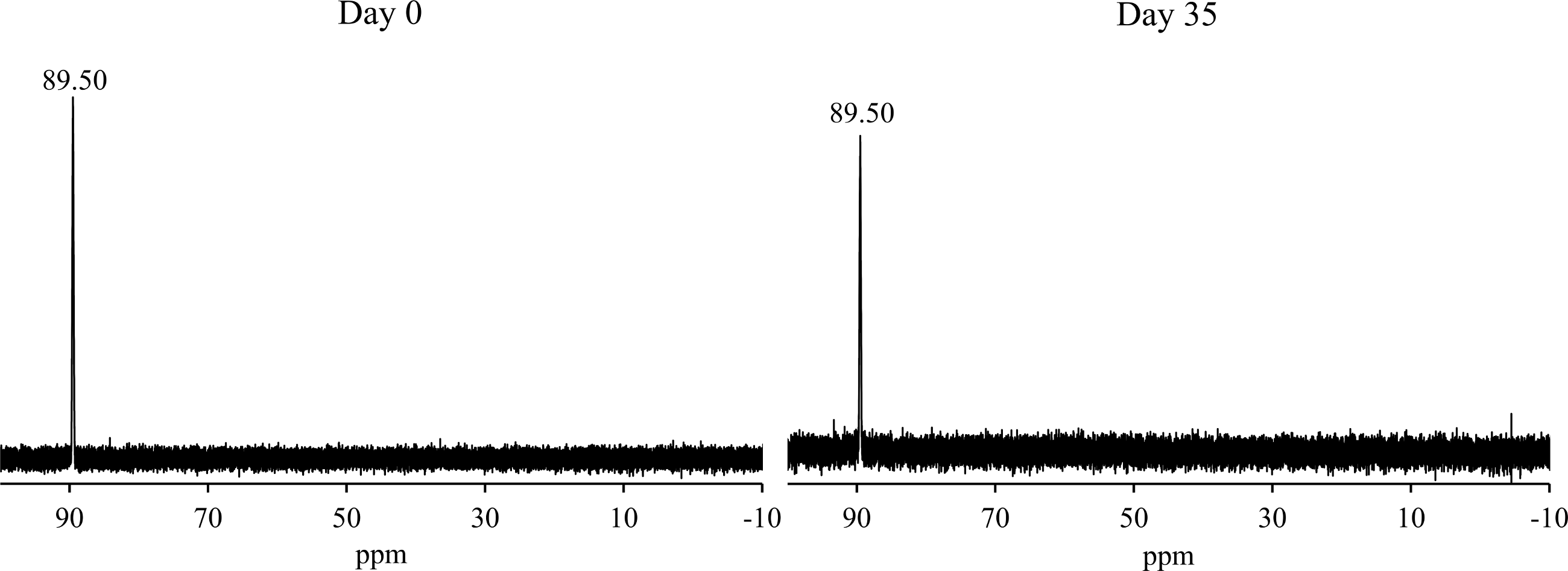

Supplement: S6 Fig — GYY-4137 was completely dissolved at a concentration of 0.12 M in 90% H2O/D2O. The 31P NMR spectrum after 35 days showed less than 3% of the GYY-4137 hydrolyzed. (TIF) [file pone.0208732.s006.tif]

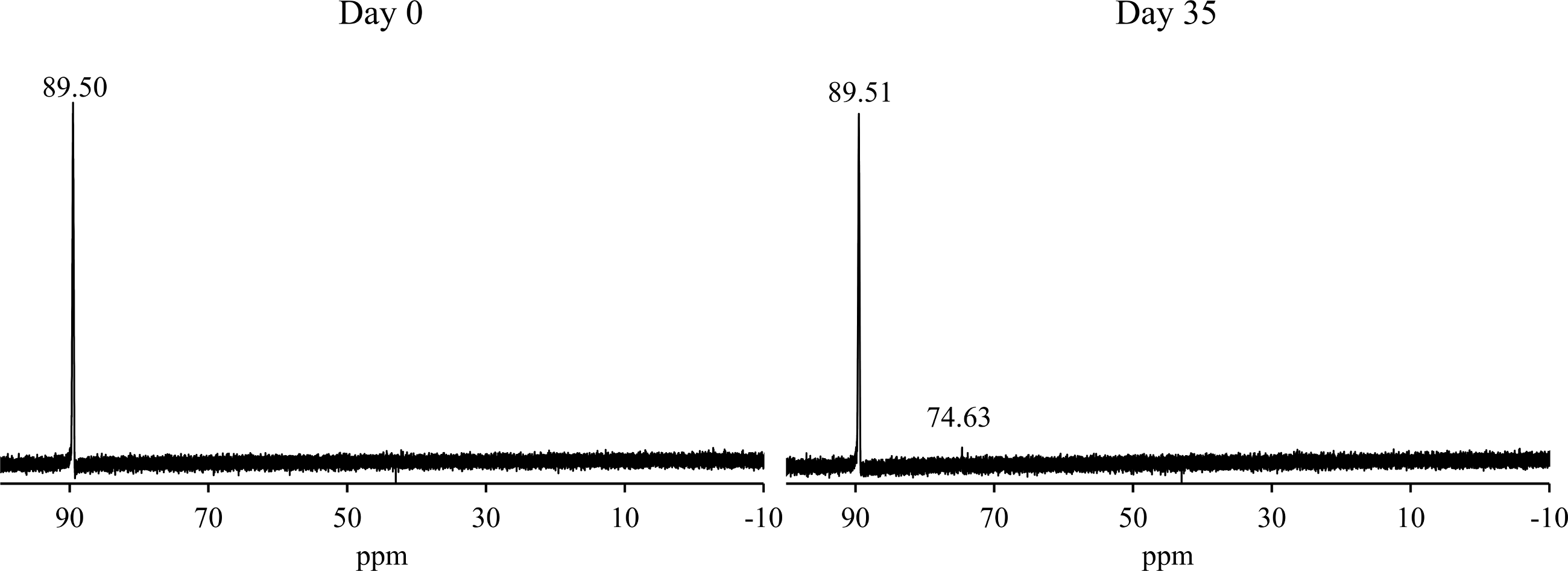

Supplement: S7 Fig — GYY-4137 was added to 90% H2O/D2O at a concentration of 0.50M. The solubility limit of GYY-4137 in water is approximately 0.13M so much of the GYY-4137 was a solid at the bottom of the NMR tube. The 31P NMR spectrum after 35 days showed less than 3% of the GYY-4137 hydrolyzed. (TIF) [file pone.0208732.s007.tif]
